# Non-tamponade Indications for Pericardial Drainage: A Case Report of Acute Streptococcus pneumoniae Purulent Pericarditis

**DOI:** 10.7759/cureus.54838

**Published:** 2024-02-24

**Authors:** Toyin Ingram, Aarti Narayan, Noah Ene, Iheoma Kwazemem- Opara, Chika Okafor

**Affiliations:** 1 Internal Medicine, Cape Fear Valley Health, Fayetteville, USA; 2 Critical Care Medicine, Cape Fear Valley Health, Fayetteville, USA

**Keywords:** non-tamponade indications, critical care cardiology, management of bacterial pericarditis, immunocompetent adult, streptococcus pneumoniae purulent pericarditis, pericardiocentesis, bacterial pericarditis

## Abstract

Pericardial drainage is a procedure completed to evacuate fluid from the pericardial space. This can be completed by pericardiocentesis or pericardial window. These procedures are most often done in the setting of cardiac tamponade, typically to correct low blood pressure due to low stroke volume from extrinsic compression of the heart chambers by the pericardial fluid. Elective pericardiocentesis can be done in cases where fluid accumulation is secondary to pathological processes, including hemopericardium secondary to complications of trauma to the chest, toxins, myocardial infarction, cardiac surgery, serosanguinous pericardial effusion due to malignancy, right heart failure, acute pericarditis, chemotherapeutic agents, metabolic derangements like uremia, and autoimmune disorders. Here, we report a case of a 66-year-old immunocompetent male with acute bacterial pericarditis resulting in fibrinous pericardial effusion without echocardiographic cardiac tamponade physiology in whom pericardial drainage proved beneficial.

## Introduction

Bacterial pericarditis is a rapidly progressive and highly fatal infection [1]. *Streptococcus pneumoniae *was previously a common cause of bacterial pericarditis; however, with the introduction of pneumococcal conjugate vaccines and antibiotics, the incidence has drastically decreased. Purulent pericarditis is the most serious manifestation of bacterial pericarditis [2]. This is characterized by gross pus in the pericardium or microscopically purulent pericardial effusion. In patients with purulent bacterial pericarditis, management includes pericardial drainage and the initiation of broad-spectrum antibiotics, which are IV vancomycin 1 g twice daily, ceftriaxone 1-2 g twice daily, and ciprofloxacin 400 mg/day [3]. Pericardial drainage is also indicated if the patient has a cardiac tamponade. However, there are cases in which a patient with purulent bacterial pericarditis can benefit from pericardial drainage without echocardiographic cardiac tamponade physiology. Here, we present a case of purulent acute *Streptococcus pneumoniae* pericarditis in a patient without echocardiographic cardiac tamponade physiology who benefited from preemptive pericardial drainage and was successfully treated with broad-spectrum antibiotics, colchicine, and high-dose aspirin.

## Case presentation

The patient was a 66-year-old male with a medical history significant for alcohol dependence and cocaine abuse. He presented to the emergency department as a code ST-elevation myocardial infarction with complaints of non-positional pleuritic chest pain and shortness of breath for three days. On arrival, vital signs were significant for blood pressure of 106/75 mmHg, respiratory rate of 31 breaths per minute, tachycardia with a heart rate of 116 beats per minute, temperature of 36.9 degrees Celsius (98.4 degrees Fahrenheit), and oxygen saturation of 99% on room air. The review of systems was significant for shortness of breath and non-positional, non-exertional, reproducible sternal chest pain. A physical exam was only significant for chest wall tenderness to palpation near the sternum. The labs included a complete blood count and comprehensive metabolic panel, as shown below in Tables 1-2, respectively. The inflammatory marker levels are shown in Table 3.

**Table 1:** The patient’s complete blood panel

Complete blood count	Reference ranges	Patient’s lab values
White blood cell count	4.5 - 12.5 x10^3^/uL	11.4 x10^3^/uL
Red blood cell count	4.70 - 6.10 x10^6^/uL	5.20 X10^6^/uL
Hemoglobin	13.5 - 18.0 g/dL	15. 1 g/dL
Hematocrit	40.5 - 54.0 %	46.4 %
Mean corpuscular volume	80.0 - 95.0 fL	89.2 fL
Platelets	150 - 450 x10^3^/uL	108 x10^3^/uL

**Table 2:** The patient’s comprehensive metabolic panel

Comprehensive metabolic panel	Reference ranges	Patient's lab values
Sodium	136 - 145 mmol/L	129 mmol/L
Potassium	3.4 - 4.9 mmol/L	4.1 mmol/L
Chloride	98 - 107 mmol/L	95 mmol/L
Carbon dioxide	21 - 32 mmol/L	20 mmol/L
Anion gap	1 - 11 mmol/L	14 mmol/L
Blood urea nitrogen	7 - 25 mg/dL	22 mg/dL
Creatinine	0.60 - 1.30 mg/dL	1.89 mg/dL
Estimated glomerular filtration rate	>60.0 mL/min/1.73m^2^	38.7 mL/min/1.73m^2^
Glucose, random	74 – 109 mg/dL	144 mg/dL
Calcium	8.6 - 10.2 mg/dL	9.1 mg/dL
Alkaline phosphatase	30 - 105 U/L	54 U/L
Albumin	3.5 - 5.7 g/dL	4.1 g/dL
Total protein	6.4 - 8.9 g/dL	7.7 g/ dL
Aspartate aminotransferase	13 - 39 U/L	18 U/L
Alanine transaminase	7 - 52 U/L	11 U/L
Bilirubin total	0.3 - 1.0 mg/dL	1.2 mg/dL
Total creatine kinase	39 - 308 U/L	94 U/L

**Table 3:** Reports showing inflammatory marker levels PCT: procalcitonin; COPD: chronic obstructive pulmonary disease

	Reference ranges	Patient's lab values
Erythrocyte sedimentation rate	0-20 mm/hr	>130 mm/hr
C-reactive protein	<5 mg/L	111 mg/L
Procalcitonin	PCT concentration <0.5 ng/ml: low risk of severe sepsis and/or septic shock; PCT concentration >2.0 ng/ml: high risk of severe sepsis and/or septic shock; PCT concentration >2.00 – <10.00: high risk for progression to severe septic shock or exacerbations of COPD; PCT concentration >/= 10.00: high likelihood of severe sepsis/septic shock or exacerbations of COPD	60.5 ng/mL

**Figure 1:**
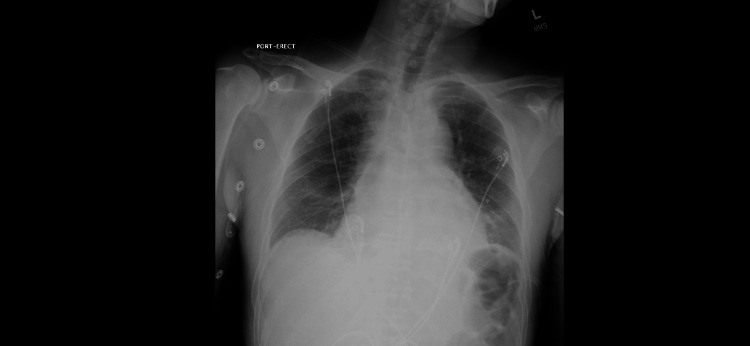
The chest X-ray showed mild pulmonary congestion and small pleural effusions.

The urine drug screen results were positive for cocaine. The electrocardiogram (EKG) showed sinus tachycardia with 1 mm ST elevations in the anterior and lateral leads, without reciprocal changes. High-sensitivity troponins test three hours apart were 18 pg/mL and 24 pg/mL, with a delta change of 6 pg/mL (reference range: 0-20 pg/mL). Bedside point-of-care ultrasound (POCUS) did not reveal any regional wall motion abnormalities. Cardiology was consulted for further evaluation. Urgent cardiac catheterization was canceled due to low suspicion of acute coronary syndrome. The patient continued to endorse pressure like chest pain, and physical examination did not show the presence of pericardial friction rub on auscultation. A repeat EKG obtained two hours after admission showed diffuse ST elevations. A formal echocardiogram revealed preserved ejection fraction (EF) and small pericardial effusions without tamponade. The patient was diagnosed with acute pericarditis with a secondary differential diagnosis of vasospastic angina secondary to cocaine use. High-dose aspirin and colchicine were initiated. The patient subsequently became acutely altered, at which time his Clinical Institute Withdrawal Assessment (CIWA) score was noted to be 13. His altered mental status was deemed secondary to his withdrawal from alcohol, and treatment was initiated with lorazepam and phenobarbital taper. Computerized tomography (CT) of the patient's head showed no intracranial abnormalities. Other workups were also non-revealing, including electrolytes, ammonia, and thyroid-stimulating hormone (TSH). Two days later, the patient had a rapid response event where he was found obtunded, tachypneic to the 50s, and unable to protect his airway. The intensive care unit (ICU) team was consulted, and he was admitted and intubated for airway protection. The patient subsequently developed a fever with a temperature of 101.4 degrees Fahrenheit. Chest X-rays (CXR) showed mild pulmonary congestion and small pleural effusions (Figure 1). There was suspicion of aspiration pneumonia or pneumonitis; hence, ceftriaxone and flagyl were initiated. Repeat CXR showed slight worsening of the right infra hilar and basilar patchy infiltrates with subtle left basilar infiltrates. Respiratory cultures and blood cultures were drawn, and he was started on a combination of piperacillin and tazobactam. Blood cultures later grew *Streptococcus pneumonia* in all four bottles. Respiratory cultures were negative. In the ICU, the patient remained hypotensive, requiring vasopressors. On day four of admission, the patient had an episode of seizure activity lasting three minutes. He was given 4 mg of lorazepam with a resolution of seizures and loaded with levetiracetam. Post-seizure, about a minute later, the patient became severely hypotensive and went into pulseless electrical activity (PEA) arrest. Chest compressions were initiated, and the return of spontaneous circulation (ROSC) was achieved after two cycles. Bedside POCUS showed worsening pericardial effusion localized near the right ventricle and right atrium. Cardiology was consulted for evaluation of possible tamponade physiology and pericardiocentesis if indicated. A detailed echocardiogram again revealed no pericardial tamponade but a significant amount of pericardial effusion over the right ventricle and right atrium, with moderate compression on the right ventricle during diastole. Fibrinous material was also noted within the pericardial effusion. The blood pressure at the time of the echocardiogram was 143/88 mmHg. A day later, a repeat echocardiogram showed a large pericardial effusion located at the right atrium and right ventricle without tamponade physiology (Video 1). Mobile echolucent material was again noted within the pericardial fluid with a dilated inferior vena cava (IVC) (Video 2). Tricuspid valve and mitral valve inflow variations were within normal limits. 

**Video 1:** A large pericardial effusion located at the right atrium and right ventricle without any signs of tamponade physiology was seen. There was mobile, echolucent material in the pericardial fluid. The right ventricle appeared to collapse in early diastole.

**Video 2:** A dilated inferior vena cava was seen.

The patient developed atrial fibrillation and rapid ventricular response (RVR), requiring an amiodarone bolus and drip as well as digoxin. Pericardiocentesis vs. pericardial window was contemplated, and pericardial window was chosen over pericardiocentesis due to the difficulty of assessing pericardial effusion via needle aspiration due to an interposed enlarged liver. The pericardial window was completed with the removal of 350 cc of purulent pericardial fluid. Pericardial tissue collected and sent for pathology results showed dense fibrous tissue with fibropurulent debris. The pericardial fluid contained mixed inflammatory cells, peripheral blood, and proteinaceous debris. The culture was negative for aerobes, anaerobes, acid-fast bacilli, fungal elements, and malignancy. 

The pericardial drain remained in place for over a month and was subsequently removed; however, the patient developed a pericardial-cutaneous fistula with continued drainage of purulent pericardial fluid. He was taken to the operating room (OR) for surgical debridement, and another drain was placed. The second drain was subsequently removed two weeks later without incident and with the resolution of bacterial pericarditis. The patient was discharged to inpatient rehab, where he continues to recover. 

## Discussion

Pericardiocentesis and pericardial window are important procedures needed for the diagnosis and management of pericardial effusions [4]. The most common indication for pericardial drainage is cardiac tamponade. Drainage of pericardial effusion is also indicated in suspected purulent and malignant pericarditis as well as chronic massive idiopathic pericardial effusion. This case reports and highlights the importance of pericardial drainage in a patient with culture-negative purulent pericardial fluid who didn't meet the typical cardiac tamponade criteria for pericardial drainage.

Acute pericarditis is diagnosed based on the presence of two out of the following four criteria: pericardial chest pain, pericardial rub, new widespread ST-segment elevation, or PR depression, and new or worsening pericardial effusion. Elevation of markers of inflammation (i.e., C-reactive protein (CRP) and erythrocyte sedimentation rate (ESR), as well as elevation of the white blood cell count) is a common and supportive finding in patients with acute pericarditis and may help monitor the activity of the disease and efficacy of therapy [4]. Pericarditis can lead to pericardial effusion, diagnosed with cardiac ultrasound. In the Western world, the majority of cases of acute pericarditis are idiopathic of viral origin, and purulent etiology accounts for 3% of cases. Other etiologies include malignancy, tuberculosis, autoimmune disorders, post-myocardial infarction, and post-cardiac surgery. Treatment of acute pericarditis includes aspirin (750-1,000 mg every eight hours (q8h) for one to two weeks) or non-steroidal anti-inflammatory drugs (NSAIDs) (e.g., ibuprofen 600 mg q8h for one to two weeks) with the addition of colchicine 0.5 mg daily (< 70 kg) or twice a day (BID) (≥ 70 kg) for three months [4].

The patient in our case presented with acute pleuritic chest pain and shortness of breath. Before making a diagnosis of acute purulent pericarditis, other diagnoses that were considered included ST-elevation myocardial infarction, pulmonary embolism, heart failure, vasospastic angina secondary to cocaine use, and community-acquired pneumonia. An ST-elevation myocardial infarction was less likely given that ST elevation was generalized rather than restricted to regional anatomic leads, and there were no reciprocal changes on the EKG. A CT angiography of the chest was negative for pulmonary embolism. A two-dimensional (2D) cardiac echo indicated normal chamber size, normal ejection fraction, normal systolic function, no valvular abnormality, and a small pericardial effusion. Hence, heart failure was ruled out. 

The patient was hemodynamically stable upon initial presentation and was managed appropriately for acute pericarditis with high-dose aspirin and colchicine. He subsequently became unstable and was transferred to the ICU, requiring vasopressors and mechanical ventilation. Blood cultures at the time grew *Streptococcus pneumoniae*. Respiratory cultures were negative. The patient was started on appropriate antibiotics. Repeat cardiac ultrasound noted worsening of pericardial effusion but without echocardiographic tamponade physiology, during which time there was a discussion as to whether the patient would benefit from pericardiocentesis or pericardial window. Eventually, the patient benefitted from a pericardial window with drainage of approximately 350 cc of purulent pericardial fluid initially and continuous pericardial drainage for eight days. The patient subsequently developed a pericardial-cutaneous fistula and completed the incision and drainage of the wound site, with the removal of approximately 300 cc of murky fluid aspirated with fluid tracking down to the pericardial space. He remained on IV Zosyn with the subsequent addition of doxycycline after the incision and drainage of the wound site. Intravenous Zosyn was switched to Unasyn and then de-escalated to Augmentin. A tissue exam collected during the pericardial window noted fibrinopurulent debris and pericardial fluid from the pericardial window, and subsequent incision and drainage was negative for acid-fast bacilli, fungi, anaerobes, and aerobes. Over a period of 18 days after the pericardial window as well as the incision and drainage of the wound site, the patient became more hemodynamically stable, likely owing to source control of infection.

Bacterial pericarditis usually occurs in one of these three ways. It could occur as a result of the spread of an infection in an abutting organ; this could be secondary to a new infection, post-surgery, or trauma. It could spread from an infected structure within the heart, for example, as seen in endocarditis. It could be a bloodborne infection that spreads through direct inoculation due to an infiltrating wound or surgical intervention [5]. The most likely source of purulent pericarditis in this case was *Streptococcus pneumoniae* bacteremia with hematogenous spread and seeding in the pericardial cavity. 

Although there are other case reports highlighting the management of cardiac tamponade and purulent pleural effusion with pericardiocentesis, this case is unique for the following reasons: the patient's drained pericardial fluid didn't grow *Streptococcus pneumoniae* or any other microorganism, despite the presence of purulent pericardial fluid requiring a pericardial window. 

## Conclusions

This case report discusses a 66-year-old male patient with a history of alcohol dependence and cocaine abuse who presented with a complex clinical scenario involving acute pericarditis, possible vasospastic angina secondary to cocaine use, alcohol withdrawal, and subsequent *Streptococcus pneumoniae* bacteremia leading to purulent pericarditis. Despite the initial absence of echocardiographic cardiac tamponade physiology, the pericardial window proved to be beneficial for this patient. This case emphasizes the importance of pericardial drainage in patients without echocardiographic tamponade. Swift diagnosis, appropriate antibiotic therapy, and, as seen in this case, pericardial drainage can be crucial in the management of such cases. This case also highlights the significance of a multidisciplinary approach involving cardiology, critical care, and infectious disease teams in the management of complex patients with multiple comorbidities and diagnostic challenges.
